# Measures matter: A scoping review of maternal and newborn indicators

**DOI:** 10.1371/journal.pone.0204763

**Published:** 2018-10-09

**Authors:** Ann-Beth Moller, Holly Newby, Claudia Hanson, Alison Morgan, Shams El Arifeen, Doris Chou, Theresa Diaz, Lale Say, Ian Askew, Allisyn C. Moran

**Affiliations:** 1 UNDP/UNFPA/UNICEF/WHO/World Bank Special Programme of Research, Development and Research Training in Human Reproduction (HRP), Department of Reproductive Health and Research, World Health Organization, Geneva, Switzerland; 2 Independent Consultant, Stockholm, Sweden; 3 Department of Public Health Sciences, Karolinska Institutet, Stockholm, Sweden; 4 Nossal Institute for Global Health, Melbourne School of Population and Global Health, University of Melbourne, Melbourne, Australia; 5 Maternal and Child Health Division, International Centre for Diarrhoeal Disease Research, Bangladesh (icddr,b), Dhaka, Bangladesh; 6 Department of Maternal, Newborn, Child and Adolescent Health World Health Organization, Geneva, Switzerland; University of South Florida, UNITED STATES

## Abstract

**Background:**

A variety of global-level monitoring initiatives have recommended indicators for tracking progress in maternal and newborn health. As a first step supporting the work of WHO’s Mother and Newborn Information for Tracking Outcomes and Results (MoNITOR) Technical Advisory Group, we aimed to compile and synthesize recommended indicators in order to document the landscape of maternal and newborn measurement and monitoring.

**Methods:**

We conducted a scoping review of indicators proposed by global multi-stakeholder groups to suggest next steps to further support maternal and newborn measurement and monitoring.

Indicators pertaining to pregnancy, childbirth, and postpartum/postnatal and newborn care were extracted and included in the indicator compilation, together with key indicator metadata. We examined patterns and relationships across the compiled indicators.

**Results:**

We identified 140 indicators linked to maternal and newborn health topics across the continuum of service provision. Fifty-five indicators relate to inputs and processes, 30 indicators relate to outputs, outcomes comprise 37 indicators in the database, and 18 impact indicators. A quarter of indicators proposed by global groups is either under development/discussion or is considered “aspirational”, highlighting the currently evolving monitoring landscape. Although considerable efforts have been made to harmonize indicator recommendations, there are still relatively few indicators shared across key monitoring initiatives and some of those that are shared may have definitional variation.

**Conclusion:**

Rapid, wide-ranging work by a number of multi-stakeholder groups has resulted in a substantial number of indicators, many of which partially overlap and many are not supported with adequate documentation or guidance. The volume of indicators, coupled with the number of initiatives promoting different indicator lists, highlight the need for strengthened coordination and technical leadership to harmonize recommendations for improved measurement and monitoring of data related to maternal and newborn heath.

## Introduction

With the adoption of the Sustainable Development Goals (SDG) [1] in 2015, countries have renewed their commitment to reduce preventable maternal and newborn deaths significantly by 2030. The SDG agenda is supported by several global initiatives and strategies such as the Global Strategy for Women’s, Children’s, and Adolescents’ Health (Global Strategy) [2], Every Newborn Action Plan (ENAP) [3], Ending Preventable Maternal Mortality (EPMM) [4], and the Global Financing Facility in Support of Every Woman Every Child [5]. These initiatives have set out goals and targets for ending preventable maternal and newborn deaths, as well as stillbirths by 2030.

Monitoring is essential for tracking progress on achieving health outcomes at global, national and sub-national levels and to ensure that investments made are leading to the anticipated improvements in health and well-being. Many indicators currently used for monitoring progress in maternal and newborn health focus on coverage of key services including antenatal care, births attended by skilled personnel, and postnatal care. These coverage indicators have been criticized as limited to measuring contact with a health provider, with little information on content or quality of care provided.[6–8] Beyond concerns about what indicators are actually being tracked, there is substantial variation in how individual indicators are defined, and how different indicators are being used across and within countries with limited coordinated guidance.[9]

In the face of these measurement challenges, a number of initiatives have been reviewing and recommending sets of indicators, as well as developing and testing novel measures and data collection methods to monitor progress in maternal and newborn health, with a focus on the content and quality of care. ENAP focuses on 10 core indicators [10], EPMM on 12 core indicators [11] and Countdown to 2030 includes 14 core indicators pertaining directly to women and newborns [12], with a number of these indicators under review or development. There is some, but not complete overlap in the indicators proposed by these initiatives, as efforts have been made to harmonize recommendations. Groups focusing specifically on maternal and newborn health have not only coordinated amongst themselves, but have also operated within larger global monitoring efforts such as the SDGs [13] and the Global Strategy [14] with an intention to inform targets and improve tracking. Similarly, coordinated measurement guidance such as that provided by the Health Data Collaborative [15] and the Global Reference List of 100 Core Health Indicators [16] has relied on the technical expertise of these expert initiatives.

The initiatives noted above primarily focus on indicators that can be tracked across a broad range of countries and are thus suitable for national and global-level monitoring. The renewed attention on tracking maternal and newborn health has stimulated thinking around how to further strengthen the evidence base; by validating indicators, further analysis of existing data, and providing better measures for tracking progress at country level, sub-nationally, and at district and facility levels where monitoring and evaluation data are essential to support decentralized planning. Recent efforts have tried to address perceived gaps. Some work has focused on routine health information system needs, such as indicators developed by the United Nations Commission on Life-Saving Commodities.[17] Several other groups, such as the IDEAS project based at the London School of Hygiene and Tropical Medicine, are conducting research on new indicators, often combining data from household and health facility surveys [8, 18, 19] or validating women’s recall of certain events around the time of birth such as the work conducted as part of Improving Coverage Measurement for Maternal Newborn and Child Health based at Johns Hopkins University[20–22], and the Quality of Care Network is developing indicators for quality improvement standards[23, 24]. Other projects, such as Transforming Newborn Measurement at the London School[25], are developing and testing indicators for program monitoring through routine health information systems.

Given the amount of ongoing work to strengthen measurement for maternal and newborn health, increased collaboration and coordination are essential as maternal and newborn health are inextricably linked. In 2016, the World Health Organization (WHO) launched the Mother and Newborn Information for Tracking Outcomes and Results (MoNITOR) Group, which functions as a technical advisory body to the WHO on matters of measurement, metrics, and monitoring of maternal and newborn health for the Departments of Maternal, Newborn, Child and Adolescent Health and Reproductive Health and Research. The purpose of MoNITOR is to provide clear, independent, harmonized, and strategic advice for global and country teams engaged in maternal and newborn measurement and accountability.[26] (Box 1).

**Box 1. Mother and Newborn Information for Tracking Outcomes and Results (MoNITOR) technical advisory group—terms of reference**

Launched in 2016, the Mother and Newborn Information for Tracking Outcomes and Results (MoNITOR) technical advisory group acts as an advisory body to WHO on matters of measurement, metrics and monitoring of maternal and newborn health for the Departments of Maternal, Newborn, Child and Adolescent Health (MCA) and Reproductive Health and Research (RHR).

The terms of reference for the MoNITOR advisory group are to:

Advise on global guidance for improving measurement, for proposed data collection on indicators relevant to maternal and newborn health.

Convene the maternal and newborn measurement community initiatives in relation to metrics, measurement and monitoring to avoid duplication of efforts and confusion in messages to the national and international communities.

Recommend priority areas related to metrics, measurement and monitoring in maternal and newborn health and how to address them.

Catalyze efforts to improve monitoring of maternal and newborn health at global and national levels especially on issues related to measurement tools, indicators and implementation of measurement guidelines.

Provide independent advice to WHO on monitoring-related guidance and norms for maternal and newborn health.

Offer advice on metrics-related research priorities and capacity building for effective implementation of monitoring and evaluation guidance and norms.

Evaluate the utility and quality of existing measurement tools, indicators and data.

As a first step to harmonizing and better defining maternal and newborn indicators, the MoNITOR advisory group recommended compiling existing maternal and newborn indicators proposed by or in use by different agencies, academic, and professional groups, including key metadata such as indicator definition, numerator and denominator, and data source. This scoping review was designed to address the research question: What is the range of indicators currently in use or recommended for global, national and subnational monitoring of maternal and newborn health?

## Materials and methods

### Study design

We adopted a scoping review design [27, 28] which was judged most adequate in view of the complexity and vast numbers of maternal and newborn indicators (PRISMA checklist see S1 Table). We compiled, mapped and categorized existing maternal and newborn indicators proposed by or reported by different agencies, academia and professional groups. The indicator compilation was conducted between February and June 2017.

### Search approach

We applied a purposive approach to identify the initiatives to include in this scoping review, with a goal of including all multi-stakeholder groups working at the global level to promote a harmonized cross-country monitoring approach with standard measures for tracking maternal and newborn health. (Box 2).

**Box 2: Global monitoring initiatives reviewed for maternal/newborn indicators**

Countdown to 2030

Every Newborn Action Plan

Ending Preventable Maternal Mortality

Global Reference List of 100 Core Health Indicators

Global Strategy for Women’s and Children’s Health

Quality, Equity, Dignity Network

Sustainable Development Goals

Technical consultation on indicators of adolescent health

United Nations Commission on Life Saving Commodities

We reviewed major global initiatives with a strong maternal and newborn health monitoring component, including the Global Strategy, ENAP, EPMM [4, 29], and Countdown to 2030. We also reviewed the Global Reference List of 100 Core Health Indicators, a major resource on health-related indicators, as well as the SDGs, as the overarching global development framework. Any indicators pertaining to pregnancy, childbirth, and postpartum/postnatal and newborn care were compiled and extracted and included in the database. Each new indicator was cross-checked against the draft database before being entered to determine whether or not it should be included as a separate indicator or whether it was a duplicate and therefore referenced accordingly.

An additional set of technical initiatives were reviewed in order to cross-check their prioritized indicators against those included in the database. These initiatives encompassed areas such as adolescent health, quality of care around the time of birth, and other maternal and newborn health projects. [23, 24, 30, 31]

A number of considerations guided decisions about the overall structure of the indicator database and the metadata. The goal of the database structure was to be concise enough to be reviewed easily but with enough detail to inform indicator harmonization. Related to this goal was how to best organize and categorize indicators to facilitate use of the database. A further consideration was the harmonization of key information, such as definitions and data source with the Global Reference List of 100 Core Health Indicators and the Global Strategy. The final structure of the database covered basic metadata such as: indicator name, indicator level on monitoring and evaluation continuum, domain, definition, numerator, denominator, disaggregation/additional dimension, feasible data sources, status of indicator (“in use”, or “under discussion/development” or “aspirational”, please see below for details) as well as definitional information and the groups using or advocating for the indicator.

### Inclusion and exclusion criteria

Given the range of aspects related to maternal and newborn health, we considered several criteria. First, the scoping review focused on indicators pertaining specifically to pregnancy, childbirth, postpartum and postnatal care, and newborn care. Second, indicators were included irrespective of the type of indicator, for example, policy or coverage indicator. Third, any indicator currently being proposed by global initiatives advocating a standardized monitoring approach, whether or not the indicator was actively in use or not, under discussion/development or if simply aspirational, was included. An important implication of this decision is there are indicators in the compilation that may be outdated or ill-defined, but they are nonetheless included because they are being advocated by a global initiative.

### Data analysis

We developed a classification system in order to be able to group the indicators and define common terms as different definitional and terminology aspects across initiatives are common.

First we grouped the indicators into the four main groups used for classification in the Global Reference List of 100 Core Health Indicators[16]: inputs and processes, outputs, outcomes, and impact.

*Inputs* are human and financial resources, physical facilities, equipment, and operational policies that enable program activities to be implemented.

*Process* refers to the multiple activities carried out to achieve the objectives of the program and include both what is done and how well it is done.

*Output* refers to the results of these efforts at the program level in terms of service access, availability, quality and safety.

*Outcome* refers to intermediate results of programs measurable at the population level, particularly service coverage.

*Impact* refers to long-term outcomes programmes are designed to affect, including decreases in mortality, morbidity and fertility.

Second part of the classification scheme was related to “current status” of the indicator by which we mean is the indicator “in use”, “under discussion/development” or “aspirational”.

We defined an indicator as “in use” if clearly defined, and is currently measured and routinely reported.

An indicator under “discussion/development” was defined as an indicator which is currently being developed.

And finally we defined an “aspirational” indicator as an indicator which requires further work to develop common definition and data collection methodologies.

A final step in the process was to examine the patterns and relationships across the compiled indicators. Specific analyses included a) the spread of indicators across a service continuum from input, through output and outcome, to impact, and b) overlap in indicators across the major maternal and newborn health monitoring initiatives.

## Results

**Fig 1:**
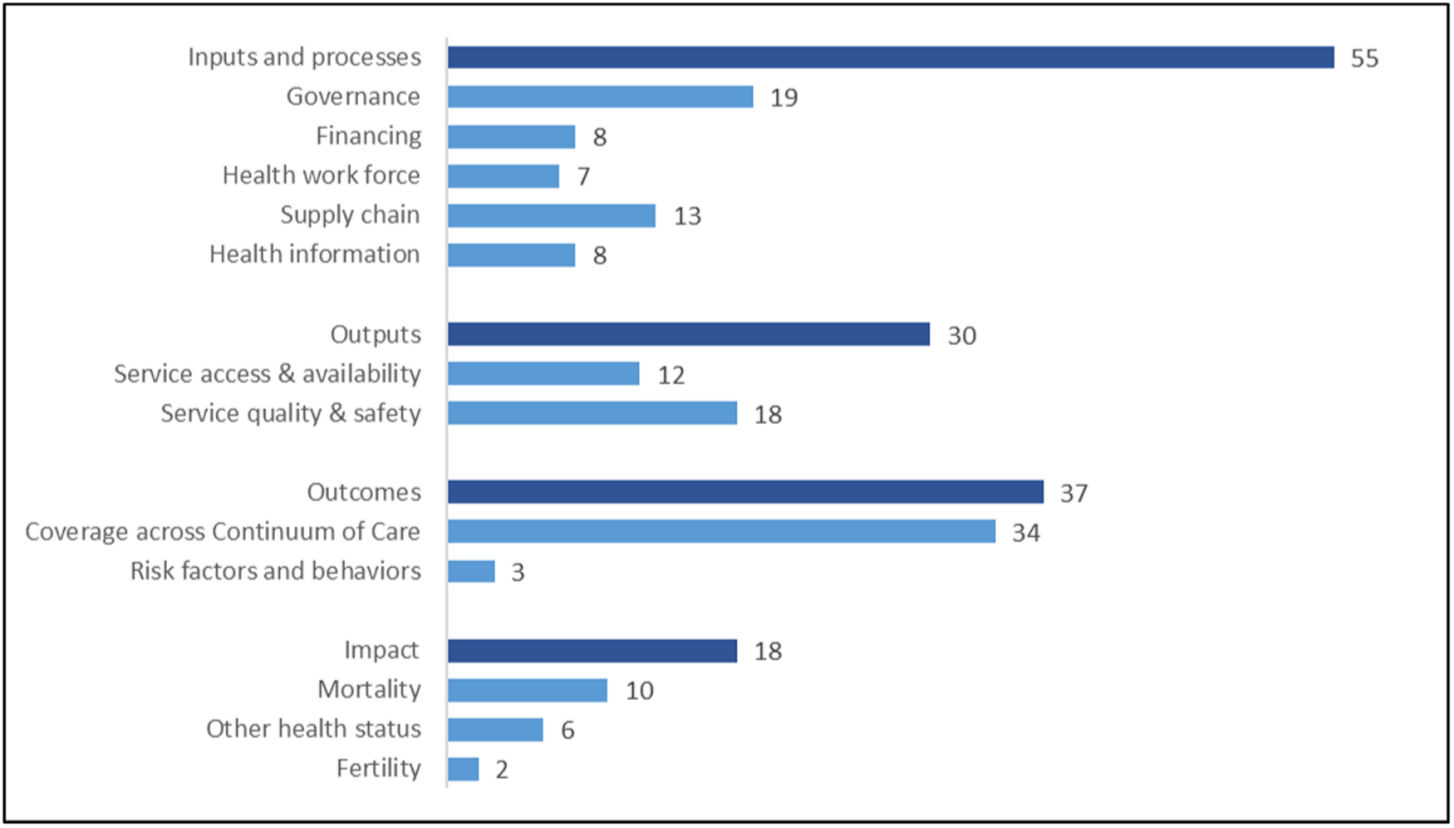
Numbers of indicators, grouped by monitoring and evaluation level and domain.

The database comprises 140 indicators linked to a variety of maternal and newborn health topics across a continuum of service provision. (S2 Table maternal and newborn indicator database) Fifty-five indicators (39%) relate to inputs and processes, such as governance and financing, the health work force, the supply chain, and health information. Thirty (21%) indicators relate to outputs, such as service access and availability, as well as service quality and safety. Outcomes, encompassing both coverage of services and health related behaviors, comprise 37 (26%) indicators in the database. A total of 18 (13%) impact indicators are included. (Table 1 and Fig 1) This breadth of indicators underscores the complexity of tracking high-quality maternal and newborn health care, and highlights the variety of data sources, from routine administrative records to household surveys, needed for monitoring from the facility level up to the national and global levels.

**Table 1:** Compiled maternal and newborn indicators by monitoring and evaluation level, and domain highlighting indicators that are currently “in use”, versus those that are “*under discussion/development”* or are “aspirational”.

Inputs and processes (55 indicators total)		Outputs (30 indicators total)	Outcomes (37 indicators total)	Impact (18 indicators total)
**Governance (19)** **1. Coordination Mechanism: A functional national coordination mechanism on RMNCH exists (or RMNCH is included in broader coordination mechanism)** **2. Maternity protection (Convention 183)** **3. International Code of Marketing of Breastmilk** **4. National policy requiring all neonatal deaths to be reviewed** **5. National policy requiring all stillbirths to be reviewed** **6. Policy on antenatal corticosteroids for preterm labor** **7. Policy on management of childbirth (MgSO4, partograph, 3rd stage)** 8. Minimum or basic newborn policy delineating the essentials of newborn care to be provided **9. Policy on chlorhexidine cord cleansing** **10. Policy on Kangaroo mother care for low birthweight newborns** 11. Presence of protocols/policies on combined care of mother and baby, immediate breastfeeding, and observations of care **12. Discharge policy (how many hours, SBA observation hours)** **13. National policy on postnatal home visits in the first week after birth** 14. Presence of Respectful Maternity Care (RMC) as a right in the national health plan(s) 15. Evidence that maternal and newborn health policies, strategies, and plans of action were formulated in coordination with other sectors **16. Civil society involvement in national maternal, newborn and child health programmes** 17. The national RMNCAH strategy/plan of action mandates community participation in decision-making, delivery of health services, and monitoring and evaluation 18. Districts/provinces have community accountability mechanisms in place to support women’s, children’s and adolescents’ health **19. Demand Generation: National RMNCH plan includes demand generation/behaviour change communication initiatives that are costed with a budget allocated** **Financing (8)** **20. Costed national implementation plan for maternal, newborn and child health** 21. Annual reviews are conducted of health spending from all financial sources, including spending on RMNCH, as part of broader health sector reviews 22. Percentage of total health expenditure spent on reproductive, maternal, newborn, and child health 23. Types of financing mechanisms for the delivery of maternal health goods and/or services identified, tested, and officially adopted **24. RMNCH expenditure by source** **25. ODA to maternal and newborn health per live birth (US$) (LSHTM method) (2013)** **26. Policy against user fees: National policy states that the life-saving commodities or related services are provided free-of-charge (i.e. no user fees) at the point of service delivery as part of essential intervention package in the public sector** **27. If fees exist for health services in the public sector, are women of reproductive age (15–49) exempt from user fees for specific services** **Health work force (7)** **28. Policy on task shifting for childbirth care** **29. Health personnel authorized for tasks and responsibilities during childbirth** **30. Midwives authorized for specific tasks** 31. Presence of a component that specifically addresses the Universal Rights of Childbearing Women (RMC Charter) in the national pre-service education curriculum for all midwifery service providers **32. Training curricula (national): In-service training curricula exist (at the national level) for interventions that deliver the commodity at the appropriate level of care** **33. Supply chain training to districts: Training in supply chain management for RMNCH commodities has been deployed to SDPs at the district level** *34. Density of midwives*, *by district (by births)*	**Supply chain (13)** **35. RMNCH plan costed and budgeted: A national RMNCH plan/strategy exists that is costed with a budget allocated for interventions that deliver LSCs at the national and sub-national levels** **36. Comprehensive national eLMIS: At the national level, there is a single electronic LMIS OR an interoperable platform for multiple LMIS that tracks commodity availability and distributions from first point of warehousing to service delivery point for each RMNCH service area AND automatically compiles and aggregates information on a continuous basis** **37. Commodity Security Strategy(ies) exist and covers the four (4) RMNC health topics including LSCs** **38. Maternal lifesaving commodities in essential medicine list** **39. Newborn lifesaving commodities in essential medicine list** **40. National Essential Medicines List (EML): Commodity is included in the national EML with a context-appropriate level of commodity specification and/or formulation** 41. Commodities included in the RMNCH costed plans 42. Whether lifesaving RMNCH commodities have products registered **43. Registered in-country: Commodity is fully registered in-country under approved & relevant formulations** **44. Results-based financing mechanism: Country entered into an agreement with the results-based financing mechanism to increase access to the life-saving commodities and related services** **45. Good Manufacturing Practices (GMP) accredited manufacturers: Procurement in the public sector is done only from manufacturers with a valid GMP accreditation certificate** 46. Availability of essential RMNCH commodities at central stores **47. Availability of antenatal corticosteroid (ACS) (%)** **Health information (8)** 48. Presence of national information system(s) that are able to record, and report data as described by ICD-PM, linking outcomes (births and deaths) to maternal and perinatal conditions, and to report annually on characteristics of births, deaths, and other vital events to produce statistics relevant to monitoring of reproductive health and mortality **49. Service utilization routinely tracked: Treatment of medical condition(s) related to the commodity are routinely tracked in a health information system (e.g. HMIS, DHIS2, LMIS)** **50. Tracked in eLMIS: Commodity availability is tracked from first point of warehousing to service delivery point by an electronic LMIS** **51. Forecasting Tools: Existence of a forecasting tool or method used routinely for forecasting needs for RMNCH medicines and medical devices** **52. Birth registration (%)** **53. Death registration coverage (%)** 54. Maternal death registration, including cause of death * 55. The maternal death surveillance and response system is reviewed annually in terms of completeness of surveillance and quality of the response, including actions to improve quality of care	**Service access and availability (12)** **1. Availability of functional EmONC facilities (per population)** **2. Met need for EmONC** 3. Availability of functional routine care: obstetric and newborn care facilities 4. Percentage of facilities that demonstrate readiness to deliver specific maternal and newborn services (%) 5. Availability of services for mothers and newborns that are provided in the same setting **6. Service-specific availability and readiness** **7. National level stock-outs: No commodity stock-out at the national level in the past 12 months** **8. Stock outs in health facilities: Percentage of point-of-service locations with commodity stock-out reported (by commodity) at time of assessment** **9. Availability of bag and mask for newborn resuscitation** **10. Availability of Kangaroo Mother Care** **11. Availability of medicine for treatment of severe neonatal infection (%)** **12. Prescription authority: Commodity prescribed at lowest appropriate level of service delivery (as per national policy and essential intervention package)** **Service quality and safety (18)** 13. Proportion of maternal and perinatal deaths and near-misses reviewed with standard audit tools (%) **14. Maternal death review coverage (%)** **15. Maternal deaths review elements** ***16. Neonatal death review coverage (%)*** **17. Facility stillbirth review (audit) in place** ***18. Institutional maternal mortality ratio (per 100 000 deliveries)*** ***19. Proportion of women who developed severe post-partum haemorrhage (PPH) (%)*** **20. National treatment guidelines exist for interventions to deliver the commodity** **21. Job aids or check lists (national): At the national level, job aids / check lists have been developed or updated for the intervention that include the specific commodity** **22. Job aids or check lists at facilities: Percentage of health facilities where relevant job aids / check lists did not exist at the facility at the time of assessment** **23. Recent training at facilities: Percentage of Health Facilities no Health Worker Trained in Service Delivery (by RMNCH component) in the past two (2) years** **24. National medicines control laboratory: At least one national medicines control laboratory exists in-country that is certified by any standards accreditation agency** **25. Medicine quality monitoring: Functioning systems exist for monitoring medicine quality in the country** **26. Patient safety monitoring: Functioning systems exist for monitoring patient safety of medicines in the country (pharmacovigilance)** **27. Proportion of maternity facilities that are "baby friendly" (%)** 28. Presence of a national grievance mechanism (ex: ombudsperson) to receive and facilitate resolution of concerns and grievances from project-affected parties related to [SRMNCAH] 29. Measure of respectful maternity care (client experience of care) 30. Antenatal, intrapartum and postpartum quality of care, including satisfaction with services received	**Service coverage (34)** **1. Antenatal care (at least one visit) (%)** **2. Antenatal care (at least four visits) (%)** *3. Antenatal care (eight or more visits) (%)* **4. Timing of first antenatal visit (%)** **5. Antenatal care content** **6. Antenatal care: blood pressure measured (%)** **7. Iron and folic acid supplements for pregnant women (%)** **8. Neonatal tetanus protection** **9. Antenatal care: tested for syphilis (%)** **10. Antenatal care: treated for syphilis (%)** **11. Antenatal corticosteroid use (%)** **12. Pregnant women counselled and tested for HIV (%)** **13. Treatment of pregnant women living with HIV (%)** **14. HIV+ pregnant women receiving ARVs for PMTCT (%)** **15. Intermittent preventive therapy for malaria during pregnancy (IPTp) (%)** **16. Births attended by skilled health personnel (%)** **17. Institutional delivery (%)** **18. Caesarean section rate** **19. Babies weighed at birth (%)** **20. Newborns receiving essential newborn care (%)** **21. Chlorhexidine cord cleansing (%)** **22. Newborns receiving thermal care (%)** ***23. Newborn resuscitation (%)*** ***24. Treatment for neonatal sepsis (%)*** **25. Kangaroo Mother Care (%)** **26. Prevention of postpartum haemorrhage in health facilities (%)** **27. Treatment of severe systemic infection/sepsis in the postnatal period (%)** ***28. Care of small and sick newborns*** **29. Early postnatal care contact for mothers and infants (%)** **30. Postnatal contact (newborns) (%)** **31. Postnatal/postpartum contact (women) (%)** *32. Postnatal quality of care* 33. Mothers who received counselling, support or messages on optimal breastfeeding at least once in the last year (%) **34. Coverage Rate: % of affected population with specified medical condition receiving treatment with appropriate life-saving commodity** **Risk factors and behaviors (3)** **35. Early initiation of breastfeeding (%)** **36. Prelacteal feeds (%)** **37. Exclusive breastfeeding rate in infants 0–5 months of age (%)**	**Mortality (10)** **1. Maternal mortality ratio (per 100 000 live births)** **2. Total maternal deaths** **3. Lifetime risk of maternal deaths** **4. Maternal cause of death** **5. Maternal near miss ratio** ***6. Percentage of maternal deaths among adolescents*** **7. Neonatal mortality rate (per 1000 live births)** **8. Neonatal deaths, as % of all <5 deaths** **9. Causes of newborn deaths** **10. Stillbirth rate (per 1000 total births)** **Other health status (6)** **11. Preterm birth rate** **12. Low birth weight among newborns, incidence** **13. Small for gestational age, prevalence** 14. Maternal morbidity rates ***15. Neonatal morbidity rates*** ***16. Disability after neonatal conditions*** **Fertility (2)** **17. Adolescent birth rate (per 1000 girls aged 10–14 year; aged 15–19 years)** **18. Percentage of women aged 20–24 years who gave birth before age 18 (%)**

A total of 102 out of the 140 indicators included in the database were classified as “in use”, thus they had a clear definition and are being used and measured by at least one global initiative. (Table 2).

**Table 2:** MNH indicators currently in use which are measured in high and low-middle income countries.

Indicators currently in use	Indicator level	Domain	Measured^1^ in high income countries^2^	Measured in low and middle income countries^2^
**Coordination Mechanism: A functional national coordination mechanism on RMNCH exists (or RMNCH is included in broader coordination mechanism)**	**Input**	**Governance**	**X**	**X**
**Maternity protection (Convention 183)**	**Input**	**Governance**	**X**	**X**
**International Code of Marketing of Breastmilk Substitutes**	**Input**	**Governance**	**X**	**X**
**National policy requiring all neonatal deaths to be reviewed**	**Input**	**Governance**	**X**	**X**
**National policy requiring all stillbirths to be reviewed**	**Input**	**Governance**	**X**	**X**
**Policy on antenatal corticosteroids for preterm labour**	**Input**	**Governance**	**X**	**X**
**Policy on management of childbirth (MgSO4, partograph, 3rd stage)**	**Input**	**Governance**	**X**	**X**
**Policy on chlorhexidine cord cleansing**	**Input**	**Governance**	**X**	**X**
**Policy on Kangaroo mother care for low birthweight newborns**	**Input**	**Governance**	**X**	**X**
**Discharge policy (how many hours, SBA observation hours)**	**Input**	**Governance**	**X**	**X**
**National policy on postnatal home visits in the first week after birth**	**Input**	**Governance**	**X**	**X**
**Civil society involvement in national maternal, newborn and child health programmes**	**Input**	**Governance**	**X**	**X**
**Demand Generation: National RMNCH plan includes demand generation/behaviour change communication initiatives that are costed with a budget allocated**.	**Input**	**Governance**	**X**	**X**
**Costed national implementation plan for maternal, newborn and child health**	**Input**	**Financing**	**X**	**X**
**RMNCH expenditure by source**	**Input**	**Financing**	**X**	
**ODA to maternal and newborn health per live birth (US$) (LSHTM method) (2013)**	**Input**	**Financing**		**X**
**Policy against user fees: National policy states that the life-saving commodities or related services are provided free-of-charge (i.e. no user fees) at the point of service delivery as part of essential intervention package in the public sector**	**Input**	**Financing**	**X**	**X**
**If fees exist for health services in the public sector, are women of reproductive age (15–49) exempt from user fees for specific services**	**Input**	**Financing**	**X**	**X**
**Policy on task shifting for childbirth care**	**Input**	**Health work force**	**X**	**X**
**Health personnel authorized for tasks and responsibilities during childbirth**	**Input**	**Health work force**	**X**	**X**
**Midwives authorized for specific tasks**	**Input**	**Health work force**	**X**	**X**
**Training curricula (national): In-service training curricula exist (at the national level) for interventions that deliver the commodity at the appropriate level of care**	**Input**	**Health work force**	**X**	**X**
**Supply chain training to districts: Training in supply chain management for RMNCH commodities has been deployed to SDPs at the district level**	**Input**	**Health work force**	**X**	**X**
**RMNCH plan costed and budgeted: A national RMNCH plan/strategy exists that is costed with a budget allocated for interventions that deliver LSCs at the national and sub-national levels**	**Input**	**Supply chain**		**X**
**Comprehensive national eLMIS: At the national level, there is a single electronic LMIS OR an interoperable platform for multiple LMIS that tracks commodity availability and distributions from first point of warehousing to service delivery point for each RMNCH service area AND automatically compiles and aggregates information on a continuous basis**	**Input**	**Supply chain**		**X**
**Commodity Security Strategy(ies) exist and covers the four (4) RMNC health topics including LSCs**	**Input**	**Supply chain**		**X**
**Maternal lifesaving commodities in essential medicine list**	**Input**	**Supply chain**		**X**
**Newborn lifesaving commodities in essential medicine list**	**Input**	**Supply chain**		**X**
**National Essential Medicines List (EML): Commodity is included in the national EML with a context-appropriate level of commodity specification and/or formulation**	**Input**	**Supply chain**		**X**
**Registered in-country: Commodity is fully registered in-country under approved & relevant formulations**	**Input**	**Supply chain**		**X**
**Results-based financing mechanism: Country entered into an agreement with the results-based financing mechanism to increase access to the life-saving commodities and related services**	**Input**	**Supply chain**		**X**
**Good Manufacturing Practices (GMP) accredited manufacturers: Procurement in the public sector is done only from manufacturers with a valid GMP accreditation certificate**	**Input**	**Supply chain**		**X**
**Availability of antenatal corticosteroid (ACS)**	**Input**	**Supply chain**		**X**
**Service utilization routinely tracked: Treatment of medical condition(s) related to the commodity are routinely tracked in a health information system (e.g. HMIS, DHIS2, LMIS)**	**Input**	**Health Information**		**X**
**Tracked in eLMIS: Commodity availability is tracked from first point of warehousing to service delivery point by an electronic LMIS**	**Input**	**Health Information**		**X**
**Forecasting Tools: Existence of a forecasting tool or method used routinely for forecasting needs for RMNCH medicines and medical devices**	**Input**	**Health Information**		**X**
**Birth registration (%)**	**Input**	**Health Information**	**X**	**X**
**Death registration coverage (%)**	**Input**	**Health Information**	**X**	**X**
**Availability of functional EmONC facilities (per population)**	**Output**	**Service access and availability**		**X**
**Met need for EmONC**	**Output**	**Service access and availability**		**X**
**Service-specific availability and readiness**	**Output**	**Service access and availability**		**X**
**National level stock-outs: No commodity stock-out at the national level in the past 12 months**	**Output**	**Service access and availability**		**X**
**Stock outs in health facilities: Percentage of point-of-service locations with commodity stock-out reported (by commodity) at time of assessment**	**Output**	**Service access and availability**		**X**
**Availability of bag and mask for newborn resuscitation**	**Output**	**Service access and availability**		**X**
**Availability of Kangaroo Mother Care**	**Output**	**Service access and availability**	**X**	**X**
**Availability of medicine for treatment of severe neonatal infection**	**Output**	**Service access and availability**		**X**
**Prescription authority: Commodity prescribed at lowest appropriate level of service delivery (as per national policy and essential intervention package)**	**Output**	**Service access and availability**		**X**
**Maternal death review coverage (%)**	**Output**	**Service quality and safety**	**X**	**X**
**Maternal deaths review elements**	**Output**	**Service quality and safety**	**X**	**X**
**Facility stillbirth review (audit) in place**	**Output**	**Service quality and safety**	**X**	**X**
**National treatment guidelines exist for interventions to deliver the commodity**	**Output**	**Service quality and safety**		**X**
**Job aids or check lists (national): At the national level, job aids / check lists have been developed or updated for the intervention that include the specific commodity**	**Output**	**Service quality and safety**		**X**
**Job aids or check lists at facilities: Percentage of health facilities where relevant job aids/check lists did not exist at the facility at the time of assessment**	**Output**	**Service quality and safety**		**X**
**Recent training at facilities: Percentage of Health Facilities no Health Worker Trained in Service Delivery (by RMNCH component) in the past two (2) years**	**Output**	**Service quality and safety**		**X**
**National medicines control laboratory: At least one national medicines control laboratory exists in-country that is certified by any standards accreditation agency**	**Output**	**Service quality and safety**		**X**
**Medicine quality monitoring: Functioning systems exist for monitoring medicine quality in the country**	**Output**	**Service quality and safety**		**X**
**Patient safety monitoring: Functioning systems exist for monitoring patient safety of medicines in the country (pharmacovigilance)**	**Output**	**Service quality and safety**	**X**	**X**
**Proportion of maternity facilities that are "baby friendly" (%)**	**Output**	**Service quality and safety**	**X**	**X**
**Antenatal care (at least one visit) (%)**	**Outcome**	**Service coverage**	**X**	**X**
**Antenatal care (at least four visits) (%)**	**Outcome**	**Service coverage**	**X**	**X**
**Timing of first antenatal visit (%)**	**Outcome**	**Service coverage**	**X**	**X**
**Antenatal care: blood pressure measured (%)**	**Outcome**	**Service coverage**		**X**
**Iron and folic acid supplements for pregnant women (%)**	**Outcome**	**Service coverage**		**X**
**Neonatal tetanus protection**	**Outcome**	**Service coverage**	**X**	**X**
**Antenatal care: tested for syphilis (%)**	**Outcome**	**Service coverage**	**X**	**X**
**Antenatal care: treated for syphilis (%)**	**Outcome**	**Service coverage**	**X**	**X**
**Antenatal corticosteroid use (%)**	**Outcome**	**Service coverage**		**X**
**Pregnant women counselled and tested for HIV (%)**	**Outcome**	**Service coverage**	**X**	**X**
**Treatment of pregnant women living with HIV (%)**	**Outcome**	**Service coverage**	**X**	**X**
**HIV+ pregnant women receiving ARVs for PMTCT (%)**	**Outcome**	**Service coverage**	**X**	**X**
**Intermittent preventive therapy for malaria during pregnancy (IPTp) (%)**	**Outcome**	**Service coverage**		**X**
**Births attended by skilled health personnel (%)**	**Outcome**	**Service coverage**	**X**	**X**
**Institutional delivery (%)**	**Outcome**	**Service coverage**	**X**	**X**
**Caesarean section rate**	**Outcome**	**Service coverage**	**X**	**X**
**Babies weighed at birth (%)**	**Outcome**	**Service coverage**	**X**	**X**
**Newborns receiving essential newborn care (%)**	**Outcome**	**Service coverage**		**X**
**Chlorhexidine cord cleansing (%)**.	**Outcome**	**Service coverage**		**X**
**Newborns receiving thermal care (%)**	**Outcome**	**Service coverage**		**X**
**Kangaroo Mother Care (%)**	**Outcome**	**Service coverage**	**X**	**X**
**Prevention of postpartum haemorrhage in health facilities (%)**	**Outcome**	**Service coverage**		**X**
**Treatment of severe systemic infection/sepsis in the postnatal period (%)**	**Outcome**	**Service coverage**		**X**
**Early postnatal care contact for mothers and infants (%)**	**Outcome**	**Service coverage**	**X**	**X**
**Postnatal contact (newborns) (%)**	**Outcome**	**Service coverage**	**X**	**X**
**Postnatal/postpartum contact (women) (%)**	**Outcome**	**Service coverage**	**X**	**X**
**Coverage Rate: % of affected population with specified medical condition receiving treatment with appropriate life-saving commodity**	**Outcome**	**Service coverage**		**X**
**Early initiation of breastfeeding (%)**	**Outcome**	**Risk factors and behaviours**	**X**	**X**
**Prelacteal feeds (%)**	**Outcome**	**Risk factors and behaviours**	**X**	**X**
**Exclusive breastfeeding rate in infants 0–5 months of age (%)**	**Outcome**	**Risk factors and behaviours**	**X**	**X**
**Maternal mortality ratio (per 100 000 live births)**	**Impact**	**Mortality**	**X**	**X**
**Total maternal deaths**	**Impact**	**Mortality**	**X**	**X**
**Lifetime risk of maternal deaths**	**Impact**	**Mortality**	**X**	**X**
**Maternal cause of death**	**Impact**	**Mortality**	**X**	**X**
**Maternal near miss ratio**	**Impact**	**Mortality**	**X**	**X**
**Neonatal mortality rate (per 1000 live births)**	**Impact**	**Mortality**	**X**	**X**
**Neonatal deaths, as % of all <5 deaths**	**Impact**	**Mortality**	**X**	**X**
**Causes of newborn deaths**	**Impact**	**Mortality**	**X**	**X**
**Stillbirth rate (per 1000 total births)**	**Impact**	**Mortality**	**X**	**X**
**Preterm birth rate**	**Impact**	**Other health status**	**X**	**X**
**Low birth weight among newborns, incidence**	**Impact**	**Other health status**	**X**	**X**
**Small for gestational age, prevalence**	**Impact**	**Other health status**	**X**	**X**
**Adolescent birth rate (per 1000 girls aged 10–14 year; aged 15–19 years)**	**Impact**	**Fertility**	**X**	**X**
**Percentage of women aged 20–24 years who gave birth before age 18 (%)**	**Impact**	**Fertility**	**X**	**X**

^1^. An indicator in considered in "use” if clearly defined and is currently measured and routinely reported.

^2^. World Bank Income grouping: https://datahelpdesk.worldbank.org/knowledgebase/articles/906519-world-bank-country-and-lending-groups.

An additional 14 indicators were classified as “under discussion/development” therefore work to advance the indicator is ongoing, for example refining the definition or testing the indicator with an aim to producing a robust measure that can be widely used. Finally, 24 indicators were considered “aspirational,” meaning a group or initiative has proposed an indicator to fill a perceived gap but there has been no significant effort to develop the indicator as yet. Notably, almost all of the aspirational variables being proposed were related to inputs and processes and as well as to outputs (16 and 6, respectively, out of 24). (Table 3).

**Table 3:** MNH indicators under development, discussion or aspirational.

*Indicators under development*	*Indicator level*	*Domain*
*Density of midwives*, *by district (by births)*	*Input*	*Health work force*
*Antenatal care content*	*Outcome*	*Service coverage*
*Newborn resuscitation (%)*	*Outcome*	*Service coverage*
*Treatment for neonatal sepsis (%)*	*Outcome*	*Service coverage*
*Care of small and sick newborns*	*Outcome*	*Service coverage*
*Postnatal quality of care*	*Outcome*	*Service coverage*
*Percentage of maternal deaths among adolescents*	*Impact*	*Mortality*
*Neonatal morbidity rates*	*Impact*	*Other health status*
*Indicators under discussion*	*Indicator level*	*Domain*
*Proportion of maternal and perinatal deaths and near-misses reviewed with standard audit tools (%)*	*Output*	*Service quality and safety*
*Neonatal death review coverage (%)*	*Output*	*Service quality and safety*
*Institutional maternal mortality ratio (per 100 000 deliveries)*	*Output*	*Service quality and safety*
*Proportion of women who developed severe post-partum haemorrhage (PPH) (%)*	*Output*	*Service quality and safety*
*Antenatal care (eight or more visits) (%)*	*Outcome*	*Service coverage*
*Disability after neonatal conditions*	*Impact*	*Other health status*
Aspirational indicators	Indicator level	Domain
Minimum or basic newborn policy delineating the essentials of newborn care to be provided	Input	Governance
Presence of protocols/policies on combined care of mother and baby, immediate breastfeeding, and observations of care	Input	Governance
Presence of Respectful Maternity Care (RMC) as a right in the national health plan(s)	Input	Governance
Evidence that maternal and newborn health policies, strategies, and plans of action were formulated in coordination with other sectors	Input	Governance
The national RMNCAH strategy/plan of action mandates community participation in decision-making, delivery of health services, and monitoring and evaluation	Input	Governance
Districts/provinces have community accountability mechanisms in place to support women’s, children’s and adolescents’ health	Input	Governance
Annual reviews are conducted of health spending from all financial sources, including spending on RMNCH, as part of broader health sector reviews	Input	Financing
Percentage of total health expenditure spent on reproductive, maternal, newborn, and child health	Input	Financing
Types of financing mechanisms for the delivery of maternal health goods and/or services identified, tested, and officially adopted	Input	Financing
Presence of a component that specifically addresses the Universal Rights of Childbearing Women (RMC Charter) in the national pre-service education curriculum for all midwifery service providers	Input	Health work force
Commodities included in the RMNCH costed plans	Input	Supply chain
Whether lifesaving RMNCH commodities have products registered	Input	Supply chain
Availability of essential RMNCH commodities at central stores	Input	Supply chain
Presence of national information system(s) that are able to record, and report data as described by ICD-PM, linking outcomes (births and deaths) to maternal and perinatal conditions, and to report annually on characteristics of births, deaths, and other vital events to produce statistics relevant to monitoring of reproductive health and mortality	Input	Health Information
Maternal death registration, including cause of death	Input	Health Information
The maternal death surveillance and response system is reviewed annually in terms of completeness of surveillance and quality of the response, including actions to improve quality of care	Input	Health Information
Availability of functional routine care: obstetric and newborn care facilities	Output	Service access and availability
Percentage of facilities that demonstrate readiness to deliver specific maternal and newborn services (%)	Output	Service access and availability
Availability of services for mothers and newborns that are provided in the same setting	Output	Service access and availability
Presence of a national grievance mechanism (ex: ombudsperson) to receive and facilitate resolution of concerns and grievances from project-affected parties related to [SRMNCAH]	Output	Service quality and safety
Measure of respectful maternity care (client experience of care)	Output	Service quality and safety
Antenatal, intrapartum and postpartum quality of care, including satisfaction with services received	Output	Service quality and safety
Mothers who received counselling, support or messages on optimal breastfeeding at least once in the last year (%)	Outcome	Service coverage
Maternal morbidity rates	Impact	Other health status

Although we categorized the indicators into these three distinct sets, the reality is more complex. A number of indicators that are categorized as “in use” may have variation in numerators and particularly denominators across initiatives, for example, or may be under active review such as being the subject of a validation study this applies currently to for example to postnatal and postpartum care indicators.

Looking across the compiled indicators highlights fragmentation in measurement efforts (Table 1). Indicators pertaining to commodities, for example, do not appear to be well-harmonized across initiatives. Some of these indicators are formulated in a generic way so that the same measurement approach could be used to track different target commodities. Other indicators, however, have been formulated to track a specific commodity. Furthermore, in terms of specific commodities needed for provision of services included in the coverage indicators, there is not always a clear link back to indicators relating to policies, supply chain, or commodity availability at the facility level that are preconditions for providing the intervention.

**Fig 2:**
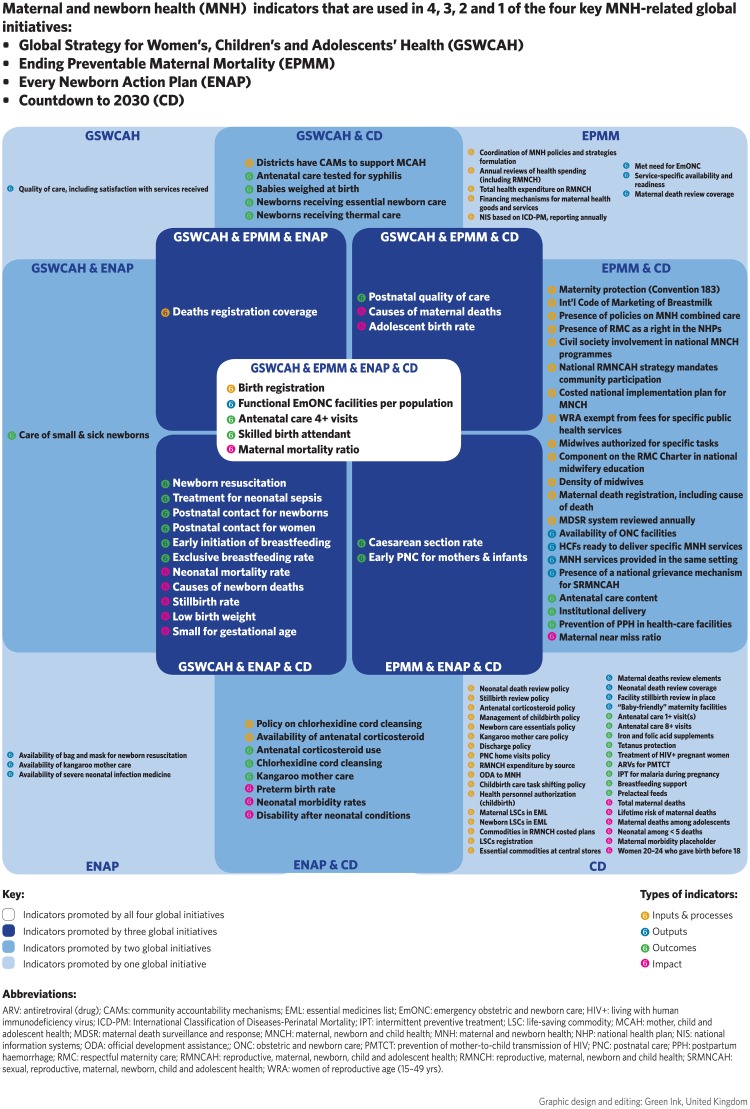
Maternal and newborn health (MNH) indicators that are used in 4, 3, 2 and 1 of the four key MNH-related global initiatives:
Global Strategy for Women’s, Children’s and Adolescents’ Health (GSWCAH)Ending Preventable Maternal Mortality (EPMM)Every Newborn Action Plan (ENAP)Countdown to 2030 (CD).

Analysis of the indicators included in just four of the major global health monitoring initiatives–the Global Strategy, ENAP, EPMM, and Countdown—provides insight into recent harmonization efforts. A total of 57 indicators out of the 140 in the database are promoted by at least two of the four initiatives. Of these, 22 indicators are promoted by at least three of the four initiatives, the majority (19) related to outcomes or impact. Just two indicators promoted by at least three initiatives are related to health information (birth registration and death registration) and one indicator relates to service access (availability of functional Emergency Obstetric and Newborn Care—EmONC). (Fig 2).

The results of the scoping review also highlight areas of divergence across the major global health monitoring initiatives. First, overlaps aside, these initiatives have different sets of indicators and even shared indicators may be prioritized differently; for example, what is considered “core” versus “additional”? Second, even some indicators that are common across initiatives may vary in terms of suggested measurement methodology. In the case of postnatal/postpartum care, for example, ENAP, EPMM and Countdown have different approaches to compute the indicators, advocating for either an indicator measuring a combined visit for mother and newborn versus separate indicators for each.

## Discussion

Political engagement, financial investments, and technical innovation have resulted in the substantial advancement of the maternal-newborn health evidence base in recent years. There has been increasing consensus around sets of standard indicators, support for data collection and data use, and efforts to address topical gaps, develop innovative methods to further analyze existing data, and to review and validate existing indicators. These advancements not only feed back into higher-level advocacy at the global level, but also serve countries by providing national health planners with a range of options from which to select indicators addressing their specific context.

As positive as these developments have been, the rapid, wide-ranging work by the different groups has resulted in a substantial number of indicators, many of which partially overlap and may not be supported with adequate documentation or guidance or with a feasible data collection platform established. The volume of indicators, coupled with the number of initiatives promoting different indicator lists, is clearly overwhelming to those who need to decide on effective monitoring and evaluation systems at national and subnational levels.

The start of a new SDG global development agenda has marked an unprecedented push to review guidelines and address data gaps, not only in terms of specific SDG indicators but encompassing a broader range of dimensions of well-being. This scoping review has been a critical first step in strengthening measurement harmonization and standardized guidance, although a number of limitations must be acknowledged. Conceptualizing and populating the database was challenging, given the broad range of factors related to maternal and newborn health, as well as the range of actors and initiatives.

Thus, decisions had to be made to limit the focus. The database aims to summarize recommendations by large-scale initiatives that have received global attention and so there are numerous technical working groups focused on the development of standard measures, such as the Chlorhexidine Working Group.[32] Furthermore, this scoping review is an imperfect reflection of even well-known monitoring initiatives. Since the scoping review was undertaken during the first half of 2017, there has been continuing work on quality of care, adolescent health, and even specific sectors of maternal and newborn care, such as antenatal care. This work will necessarily inform revisions in monitoring recommendations. Finally, inconsistencies in naming and definition conventions across initiatives made it challenging at times to determine whether a single indicator or separate indicators should be listed in the database.

Thus, the database is not intended as a definitive list of indicators. Rather, it is designed to be a tool that can help inform the next steps to advance effective maternal-newborn health monitoring by broadly depicting the current measurement landscape. Although the database is an ongoing effort that will continue to be refined and populated, it is nonetheless informative about which indicators are being advocated, what gaps remain, and whether further methodological work is still needed. As most of the research and indicator development has been undertaken by global initiatives there remains a gap in what measures are most useful for individual national and subnational contexts.

Our mapping presents thus a first step and will need to be followed by a set of actions such as to harmonize definitions, address measurement issues and gaps, select a smaller set of core indicators, and propose indicators for which investment and research is needed. More investments are in particular needed to develop guidance on indicators beyond the well-established impact and outcome indicators, and data collection tools including suggestions for maximizing use of all data sources [33]. The results of the scoping review can also guide thinking around addressing monitoring needs at the national and subnational levels by highlighting a series of technical areas in need of strengthening, including, a searchable indicator database made available to the public, technical guidance on key indicators, country level guidance for indicator selection and prioritization, country level guidance on development of HMIS and registries to better capture MNH, and research that operationalizes aspirational indicators. The MoNITOR group is currently drafting a research protocol that will support a series of country case studies to address these gaps and assess the need for country specific support and monitoring guidance.

## Conclusion

This scoping review forms a ‘stock take’ of current maternal and newborn indicators. The next steps include documenting the validation gaps and measurement challenges inherent in many of the existing indicators, harmonizing the indicator definition and proposing a set of core indicators and developing indicator specific guidance sheets.

## Supporting information

S1 Table PRISMA 2009 checklist. (DOC)

S2 Table Maternal and newborn indicator database. (XLSX)
