# Supplementary material for: Measures matter: A scoping review of maternal and newborn indicators
Source: PLoS One. 2018 Oct 9;13(10):e0204763. doi: 10.1371/journal.pone.0204763 (PMC6177145; doi:10.1371/journal.pone.0204763)
Supplement: S1 Table — (DOC) [file pone.0204763.s001.doc]

| **Section/topic** | **#** | **Checklist item** | **Reported on page #** |
| --- | --- | --- | --- |
| **TITLE** | | |  |
| Title | 1 | Identify the report as a systematic review, meta-analysis, or both. | N/A (scoping review) |
| **ABSTRACT** | | |  |
| Structured summary | 2 | Provide a structured summary including, as applicable: background; objectives; data sources; study eligibility criteria, participants, and interventions; study appraisal and synthesis methods; results; limitations; conclusions and implications of key findings; systematic review registration number. | 2 (Background, Methods, Results, Conclusion) |
| **INTRODUCTION** | | |  |
| Rationale | 3 | Describe the rationale for the review in the context of what is already known. | 6 |
| Objectives | 4 | Provide an explicit statement of questions being addressed with reference to participants, interventions, comparisons, outcomes, and study design (PICOS). | 6 |
| **METHODS** | | |  |
| Protocol and registration | 5 | Indicate if a review protocol exists, if and where it can be accessed (e.g., Web address), and, if available, provide registration information including registration number. | N/A |
| Eligibility criteria | 6 | Specify study characteristics (e.g., PICOS, length of follow-up) and report characteristics (e.g., years considered, language, publication status) used as criteria for eligibility, giving rationale. | 6-7 |
| Information sources | 7 | Describe all information sources (e.g., databases with dates of coverage, contact with study authors to identify additional studies) in the search and date last searched. | 6 |
| Search | 8 | Present full electronic search strategy for at least one database, including any limits used, such that it could be repeated. | N/A |
| Study selection | 9 | State the process for selecting studies (i.e., screening, eligibility, included in systematic review, and, if applicable, included in the meta-analysis). | 6-7 |
| Data collection process | 10 | Describe method of data extraction from reports (e.g., piloted forms, independently, in duplicate) and any processes for obtaining and confirming data from investigators. | 6-7 |
| Data items | 11 | List and define all variables for which data were sought (e.g., PICOS, funding sources) and any assumptions and simplifications made. | N/A (indicators only) |
| Risk of bias in individual studies | 12 | Describe methods used for assessing risk of bias of individual studies (including specification of whether this was done at the study or outcome level), and how this information is to be used in any data synthesis. | N/A |
| Summary measures | 13 | State the principal summary measures (e.g., risk ratio, difference in means). | 7-8 |
| Synthesis of results | 14 | Describe the methods of handling data and combining results of studies, if done, including measures of consistency (e.g., I^2^) for each meta-analysis. | N/A |

Page 1 of 2

| **Section/topic** | **#** | **Checklist item** | **Reported on page #** |
| --- | --- | --- | --- |
| Risk of bias across studies | 15 | Specify any assessment of risk of bias that may affect the cumulative evidence (e.g., publication bias, selective reporting within studies). | N/A |
| Additional analyses | 16 | Describe methods of additional analyses (e.g., sensitivity or subgroup analyses, meta-regression), if done, indicating which were pre-specified. | N/A |
| **RESULTS** | | |  |
| Study selection | 17 | Give numbers of studies screened, assessed for eligibility, and included in the review, with reasons for exclusions at each stage, ideally with a flow diagram. | 8-9 |
| Study characteristics | 18 | For each study, present characteristics for which data were extracted (e.g., study size, PICOS, follow-up period) and provide the citations. | N/A |
| Risk of bias within studies | 19 | Present data on risk of bias of each study and, if available, any outcome level assessment (see item 12). | N/A |
| Results of individual studies | 20 | For all outcomes considered (benefits or harms), present, for each study: (a) simple summary data for each intervention group (b) effect estimates and confidence intervals, ideally with a forest plot. | N/A |
| Synthesis of results | 21 | Present results of each meta-analysis done, including confidence intervals and measures of consistency. | N/A |
| Risk of bias across studies | 22 | Present results of any assessment of risk of bias across studies (see Item 15). | N/A |
| Additional analysis | 23 | Give results of additional analyses, if done (e.g., sensitivity or subgroup analyses, meta-regression [see Item 16]). | N/A |
| **DISCUSSION** | | |  |
| Summary of evidence | 24 | Summarize the main findings including the strength of evidence for each main outcome; consider their relevance to key groups (e.g., healthcare providers, users, and policy makers). | 10-11 |
| Limitations | 25 | Discuss limitations at study and outcome level (e.g., risk of bias), and at review-level (e.g., incomplete retrieval of identified research, reporting bias). |  |
| Conclusions | 26 | Provide a general interpretation of the results in the context of other evidence, and implications for future research. | 10-11 |
| **FUNDING** | | |  |
| Funding | 27 | Describe sources of funding for the systematic review and other support (e.g., supply of data); role of funders for the systematic review. | 12 |

*From:* Moher D, Liberati A, Tetzlaff J, Altman DG, The PRISMA Group (2009). Preferred Reporting Items for Systematic Reviews and Meta-Analyses: The PRISMA Statement. PLoS Med 6(7): e1000097. doi:10.1371/journal.pmed1000097

For more information, visit: **www.prisma-statement.org**.

Page 2 of 2

**Additional information for PONE-D-18-15212**

**Measures matter: a scoping review of maternal and newborn indicators**

1. Flowchart – This flowchart is not applicable to this scoping review as maternal and newborn indicators from different global initiatives were reviewed.

2. Full list of all sources reviewed:

1. United Nations. Sustainable Development Goals New York: United Nations Department of Economic and Social Affairs; [cited 2017 November 30]. Available from: https://sustainabledevelopment.un.org/index.html.

2. Every Woman Every Child. Global strategy for women’s, children’s and adolescents’ health 2016–2030 2015 [cited 2017 21 October]. Available from: http://globalstrategy.everywomaneverychild.org/.

3. Every Newborn: an action plan to end preventable deaths. Geneva: World Health Organization; 2014. http://www.who.int/maternal_child_adolescent/newborns/every-newborn/en/

4. Strategies towards ending preventable maternal mortality (EPMM). Geneva: World Health Organization; 2015. http://www.who.int/reproductivehealth/topics/maternal_perinatal/epmm/en/

5. Global Financing Facility in support of Every Woman Every Child (GFF) Washington, DC: World Bank Group; 2016 [cited 2017 21 October]. Available from: http://globalfinancingfacility.org/.

6. Moran AC, Jolivet RR, Chou D, Dalglish SL, Hill K, Ramsey K, et al. A common monitoring framework for ending preventable maternal mortality, 2015-2030: phase I of a multi-step process. BMC Pregnancy Childbirth. 2016;16:250.

7. Countdown to 2030: The 2017 report. New York: UNICEF, 2017. http://countdown2030.org/reports-and-publications/countdown-2017-report

9. Every Woman Every Child. Indicator and Monitoring Framework for the Global Strategy for Women’s, Children’s and Adolescent’s Health (2016–2030). Geneva: WHO; 2016. http://www.who.int/life-course/publications/gs-Indicator-and-monitoring-framework.pdf

10. Health Data Collaborative - Data for health and sustainable development [cited 2017 11 October]. Available from: https://www.healthdatacollaborative.org/.

11. World Health Organization. Global reference list of 100 core health indicators. Geneva, Switzerland: World Health Organization; 2015. http://www.who.int/healthinfo/indicators/2015/en/

12. IDEAS [cited 2017 October 13]. Available from: https://ideas.lshtm.ac.uk/.

13. £5m project to transform measurement for every newborn | LSHTM 2017 [cited 2017 December 21]. Available from: https://www.lshtm.ac.uk/newsevents/news/2015/measurement_for_every_newborn.html.

14. Maternal Health Task Force, editor Report on Ending Preventable Maternal Mortality (EPMM) Expert Meeting, Phase II: Indicators for the Social, Political, and Economic Determinants of EPMM21-22 September 2016; Cambridge, Massachusetts, USA. https://cdn2.sph.harvard.edu/wp-content/uploads/sites/32/2016/08/EPMM-Phase-II-Monitoring-Framework-Survey-Reference-Guide.pdf

15. World Health Organization. Technical consultation on indicators of adolescent health, WHO, Geneva, Switzerland, 30 September-1 October 2014. Geneva, Switzerland; 2015. http://www.who.int/maternal_child_adolescent/documents/adolescent-health-technical-consultation/en/

16. Chlorhexidine Working Group. Performance Indicators: Chlorhexidine for Umbilical Cord Care. https://www.healthynewbornnetwork.org/hnn-content/uploads/Performance-Indicators-CHX.pdfJuly 2016.

17. Pronyk PM, Nemser B, Maliqi B, Springstubb N, Sera D, Karimov R, et al. The UN Commission on Life Saving Commodities 3 years on: global progress update and results of a multicountry assessment. Lancet Glob Health. 2016;4(4):e276-86.

3. Researchers who did research and extraction:

Three researchers did the research and extraction: Holly Newby, Allisyn C Moran, and Ann-Beth Moller
